# Signatures of selection identify loci associated with milk yield in sheep

**DOI:** 10.1186/1471-2156-14-76

**Published:** 2013-09-03

**Authors:** Bianca Moioli, Maria Carmela Scatà, Roberto Steri, Francesco Napolitano, Gennaro Catillo

**Affiliations:** 1 Consiglio per la Ricerca e la sperimentazione in Agricoltura, via Salaria 31, Monterotondo 00015, Italy

**Keywords:** OvineSNP50K Beadchip, Dairy sheep, Candidate genes

## Abstract

**Background:**

Identification of genomic regions that have been targets of selection for phenotypic traits is one of the most challenging areas of research in animal genetics, particularly in livestock where few annotated genes are available. In this study a genome-wide scan using the Illumina SNP50K Beadchip was performed in the attempt to identify genomic regions associated with milk productivity in sheep. The ovine genomic regions encoding putative candidate genes were compared with the corresponding areas in Bos taurus, as the taurine genome is better annotated.

**Results:**

A total of 100 dairy sheep were genotyped on the Illumina OvineSNP50K Beadchip. The Fisher’s exact test of significance of differences of allele frequency between each pair of the two tails of the distribution of top/worse milk yielders was performed for each marker. The genomic regions where highly divergent milk yielders showed different allele frequencies at consecutive markers was extracted from the OAR v3.1 Ovine (Texel) Genome Assembly, and was compared to the corresponding areas in Bos taurus, allowing the detection of two genes, the Palmdelphin and the Ring finger protein 145. These genes encoded non-synonymous mutations correlated with the marker alleles.

**Conclusion:**

The innovation of this study was to show that the DNA genotyping with the Illumina SNP50K Beadchip allowed to detect genes, and mutations in the genes, which have not yet been annotated in the livestock under investigation.

## Background

The recent availability of sheep genome-wide SNP panels could potentially allow to identify genomic regions and hopefully mutations that underlie the desired performance. The examination of variation in SNP allele frequencies between populations is a promising strategy to identify likely targets of past selection practices in order to identify the genes underlying phenotypic variation [1]. To date, a number of examples have successfully identified genomic regions subject to positive selection in human and cattle [2-6]. Although sheep production constitutes a major proportion of agricultural output, both in the developing countries, and in Australia, Northern Europe and the Mediterranean basin, the lower monetary value of an adult sheep, compared to cattle, could not give sufficient impulse to the research either in sheep breeding or sheep genomics. This limitation has recently been reduced with the availability of the Ovine SNP50k BeadChip [7], containing approximately 50,000 SNP distributed along the whole genome, with knowledge of the position on each specific chromosome. Kijas *et al*. [8] performed a genome-wide analysis of the world’s sheep breeds and showed that particular regions of the ovine genome contained strong evidence for accelerated change in response to artificial selection. On the other hand, Moradi et. al. [7] identified selection sweeps in fat and thin tail sheep breeds.

Domesticated species provide an excellent opportunity to understand how artificial selection promotes variation in an economic trait; when a new beneficial mutation increases in frequency, because of selection, a selective sweep occurs, consisting in the elimination of variation in other genomic regions linked to that mutation [9]. For the identification of selection sweeps for milk traits, successful application of the selective genotyping strategy for QTL mapping has been reported in dairy cattle [10-12]. In these cases, the extreme divergent individuals for a trait (the two tails of the distribution) are chosen and genotyped. With the aim to identify regions of the ovine genome that potentially affect milk productivity, we genotyped with the Illumina SNP50K Beadchip a population of dairy sheep, extremely variable as regards to milk traits; similarly to the selective genotyping strategy, we examined the variation in SNP allele frequencies between the two tails of the distribution; the genomic regions surrounding or encoding the markers mostly differing in allele frequency between the distribution tails were then checked for the presence of expressed genes either in sheep or other mammals, being sheep provided by many less annotated genes than cattle.

## Results

### Genotyping and data mining

A total of 100 sheep of the Altamurana breed, having milk production records available for consecutive lactations, were genotyped on the Illumina OvineSNP50K Beadchip. Residuals of lactation milk yield of every sheep were calculated with a mixed model that included the year and the age at lambing, being those the most important source of variation of milk yield. The two tails of the distribution of milk yield residuals were used to compare the allele frequency at each marker between top and worse milk yielders. Average residuals for each group were reported in Table 1.

**Table 1:** Average milk yield residuals for divergent groups of milk yielders

**Category**	**N animals**	**Mean (l milk)**	**Std**
Top sixth	16	39.7	9.3
Worse sixth	16	−29.0	5.2
Top twelfth	8	47.2	8.1
Worse twelfth	8	−32.5	5.5

After data editing, 49,225 SNP loci genotyped in 100 individuals passed quality control and were used in the subsequent analysis. Number of markers was highest in chromosomes 1 (5387) and 2 (5030) and lowest in chromosome 24 (666) and 22 (816), but marker density was similar, ranging from 48 K bp to 61 K bp.

The Fisher’s exact test of significance of differences of allele frequency between each pair of the two tails of the distribution was performed for each marker. The markers satisfying the condition where P_1/6_ < .05; and P _1/12_ <0.001 were 52 (Table 2).

**Table 2:** Markers presenting different allele frequencies between divergent milk yielders

**Chromosome**	**Marker**	**Coordinates on OAR v3.1**
1	s53787.1	68333165
1	OAR1_81453876.1	76272189
1	OAR1_81508823.1	76328774
1	OAR1_81533009.1	76354339
1	OAR1_82011046.1	76772898
1	OAR1_82047913.1	76809785
1	OAR1_83216080.1	77946622
1	OAR1_83062340.1	77794629
1	OAR1_84322183.1	79028365
1	OAR1_84711156.1	79406401
1	OAR1_84872548.1	79569865
1	OAR1_85050388.1	79748706
1	OAR1_85487979.1	80172324
1	OAR1_85599941.1	80284491
1	OAR1_85975832.1	80705932
1	OAR1_86003731.1	80723243
1	OAR1_86737634.1	81421911
1	OAR1_86871264.1	81547416
1	OAR1_86891635.1	81567844
1	s21838.1	81719675
1	OAR1_87788428.1	82558593
1	OAR1_87905281.1	82670212
1	OAR1_88413743.1	83178835
1	OAR1_88422556.1	83187690
1	OAR1_88722215.1	83469881
1	s16601.1	85315701
1	OAR1_93124341.1	87709264
1	OAR1_94322642.1	88759553
1	OAR1_95138062.1	89509677
1	DU461361_483.1	256749808
3	OAR3_90352913.1	85412113
5	s57638.1	58909273
5	OAR5_74765699.1	68064730
5	OAR5_74795642.1	68095509
5	OAR5_74829194.1	68129433
6	OAR6_36739717.1	32655828
6	OAR6_37176737.1	33197652
6	OAR6_43343515.1	38952950
6	OAR6_43416865.1	39035619
12	s62621.1	72535317
15	OAR15_62829975.1	57409463
19	s17877.1	54653935
19	s35547.1	58530011
21	OAR21_12416673.1	10876715
22	OAR22_26886276.1	22785443
22	s60172.1	22808391
24	s36249.1	12686386
26	s33938.1	38189272
26	OAR26_46471996_X.1	41014058
26	OAR26_49141508.1	43255184
26	OAR26_49162631.1	43276441
X	OARX_124970841.1	100602007

A further assumption was that the mentioned conditions were satisfied by at least two consecutive markers, where the distance between the two was no more than 100 K bp. Twenty-one markers satisfied this condition (Table 3). It was interesting to note that, in 2, cases the condition was satisfied by three consecutive markers, encompassing 2 regions on chromosomes 1 and one region on chromosome 5 (Table 3).

**Table 3:** Consecutive markers no more than 100 Kbp distant from each other

**Chromosome**	**Marker 1**	**Distance (bp) between markers 1 and 2**	**Marker 2**	**Distance (bp) between markers 2 and 3**	**Marker 3**
1	OAR1_81453876.1	56585	OAR1_81508823.1	25565	OAR1_81533009.1
1	OAR1_82011046.1	36887	OAR1_82047913.1		
1	OAR1_85975832.1	17311	OAR1_86003731.1		
1	OAR1_86871264.1	20428	OAR1_86891635.1		
1	OAR1_88413743.1	8855	OAR1_88422556.1		
5	OAR5_74765699.1	30779	OAR5_74795642.1	33924	OAR5_74829194.1
6	OAR6_43343515.1	82669	OAR6_43416865.1		
22	OAR22_26886276.1	22948	s60172.1		
26	OAR26_49141508.1	21257	OAR26_49162631.1		

The search for expressed genes was therefore performed within the nine regions comprised between the markers of Table 3.

### Study of the expressed genes in the candidate regions

For each of the nine regions, the sequence between the markers there included was extracted from the OAR v3.1 Ovine (Texel) Genome Assembly in http://www.livestockgenomics.csiro.au. A standard nucleotide blast [13] of the ovine sequence was performed against the NCBI data base of the expressed sequence tags, allowing the detection of two bovine genes: the Palmdelphin (PALMD) in the region comprised between OAR1_81453876.1 and OAR1_81533009.1 and the Ring finger protein 145 (RFP145) in the region comprised between OAR5_74765699.1 and OAR5_74829194.1. The respective bovine mRNA sequences NM_001035079.1 for PALMD and NM_001102168.1 for RFP145 allowed the positioning of the corresponding ovine exons on the OAR v3.1 Ovine (Texel) Genome Assembly.

### Analysis of PALMD gene

The standard blast of the sequence between OAR1_81453876.1 and OAR1_81533009.1 had made evident 98% nucleotide homology with exons 6, 7 and 8 of the Bos taurus PALMD mRNA (NM_001035079.1). To confirm that that region encoded the ovine PALMD gene, further 76 K bp upstream OAR1_81453876.1 were also extracted from the OAR v3.1 Ovine (Texel) Genome Assembly, and blasted to NM_001035079.1. This region contained two markers (s43262.1 and OAR1_81421530.1) that had not been considered previously, having similar allele frequency between high and low yielding sheep. In this way, it was possible to assess the estimated position of exons 1 to 5 (Table 4), and it was also assessed that exon 5 was fully comprised in the flanking regions of OAR1_81453876.1 (Table 4).

**Table 4:** **Estimated positions of the ovine PALMD exons on OAR v3**.**1 Genome Assembly**

**Marker**	**Coordinates on OAR v3.1**	**Exons**	**Estimated position of the exons on OAR v3.1**
s43262.1	76197141		
		Exon 1	76220164 - 76220213
OAR1_81421530.1	76126901		
		Exon 2	76245910 - 76245991
		Exon 3	76252401 - 76253527
		Exon 4	76271886 - 76272005
		Exon 5	76272142 - 76272177
OAR1_81453876.1	76272189		
		Exon 6	76272297 - 76272413
		Exon 7	76274174 - 76275271
		Exon 8 + 3′ UTR	76279661 - 76280019
OAR1_81508823.1	76328774		
OAR1_81533009.1	76354339		

Assuming the existence of a putative mutation affecting milk production which should fall between OAR1_81453876.1 and OAR1_81533009.1, these markers presenting extremely different allele frequency between the tails, five amplicons were designed on the ovine sequence so to amplify exons 5 to 8 and part of the flanking introns.

Direct sequencing of the DNA of 5 homozygous sheep at OAR1_81453876.1 and OAR1_81533009.1, and 5 more homozygous for the opposite alleles, allowed to detect a non-synonymous mutation (Ser/Thr) in exon 5, 18 bp upstream OAR1_81453876.1, and a synonymous mutation in exon 7. The detected mutations were reported in Table 5. Direct sequencing of DNA of thirty more sheep at the amplicon encoding the non-synonymous mutation revealed .98 correlation of this mutation with the alleles of OAR1_81453876.1 and OAR1_81533009.1.

**Table 5:** Position and potential effects of the detected mutations in the PALMD gene

**Location in the gene**	**Accession**	**Mutation**	**Amino acid change**
Exon 5	NM_001035079	c.547 T > A	Ser/Thr
Exon 7	NM_001035079	c.1320G > A	Synonymous (Gln/Gln)

### Analysis of RFP145 gene

The standard blast [13] of the sequence between OAR5_74765699.1 and OAR5_74829194.1 showed that this genomic region encoded the RFP145 gene. In detail (Table 6), exon 1 was comprised between OAR5_74829194.1 and OAR5_74822290.1; exon 2 and 3 were comprised between OAR5_74822290.1 and OAR5_74795642.1; exon 4 to 10 and the 3′UTR were comprised between OAR5_74795642.1 and OAR5_74765699.1, being the last two markers more than 30 K bp distant from each other.

**Table 6:** **Estimated positions of the ovine RFP145 exons on OAR v3**.**1 Genome Assembly**

**Marker**	**Coordinates on OAR v3.1**	**Exons**	**Estimated position of the exons on OAR v3.1**
OAR5_74829194.1	68129433		
		Exon 1	68127463 - 68127277
OAR5_74822290.1	68122479		
		Exon 2	68117911 - 68117802
		Exon 3	68103476 - 68103390
OAR5_74795642.1	68095509		
		Exon 4	68093341 - 68093100
		Exon 5	68090335 - 68090158
		Exon 6	68084871 - 68084728
		Exon 7	68081957 - 68081773
		Exon 8	68075044 - 68074895
		Exon 9	68074013 - 68073653
		Exon 10 + 3′ UTR	68070963 - 68069349
OAR5_74765699.1	68064730		

To detect putative responsible mutations in the RFP gene affecting milk performances, 12 amplicons were designed on the ovine sequence so to amplify the ten exons and part of the flanking introns as well.

Direct sequencing was performed of the DNA of 5 GG homozygous sheep at OAR5_74829194.1 being these sheep also CC homozygous at OAR5_74765699.1; moreover, direct sequencing was performed also on the DNA of 5 more homozygous AA sheep at both OAR5_74765699.1 and OAR5_74829194.1. The detected mutations were reported in Additional file 1: two synonymous mutations in exon 2 and 4 respectively and one non-synonymous mutation in exon 10. The in silico analysis [14] made evident that two mutations, respectively in the 5′UTR and in intron 1, were located within the sequence of putative binding sites of transcription factors (Additional file 1), the second mutation falling in the core sequence of the binding factor (capital letters).

The mutation falling in intron 1 of the RFP145 gene (Additional file 1) was fully correlated with OAR5_74765699.1 and OAR5_74829194.1; as regards to the other mutations, only one of the two homozygous groups showed to be homozygous for the same allele at the novel mutation, but the other group presented either of the alleles, sometimes in a heterozygous form.

### Effect of the PALMD Ser/Thr mutation on milk yield in an independent sheep population

To demonstrate the association of the PALMD Ser/Thr mutation with milk traits, the amplicon encoding the Ser/Thr mutation was sequenced in 89 of the sheep of the original sample. It was not possible to obtain the sequence for all 100 Altamurana sheep, because 11 of them were no more in the farm, their DNA being no more available either. Moreover, to verify the hypothesis that the PALMD Ser/Thr mutation affected milk traits also in different populations than the one in which the strong LD between OAR1_81453876.1 and OAR1_81533009.1 had been detected, the amplicon encoding the Ser/Thr mutation was sequenced in 35 more sheep of the Sarda breed, the most popular Italian specialized dairy sheep. Lactation milk yield and days in milk of the two breeds were reported in Table 7. In a data set which included 256 lactations, 70 from the Sarda and 186 from the Altamurana, the effect of the Thr > Ser substitution was estimated with a linear regression of the number of A alleles (A = Thr), on lactation milk yield. The average allele substitution effect (T to A substitution) was highly significant (+ 10.1 l milk ± 4; *P* = 0.02 for the Altamurana breed and + 22.9 l milk ± 8; *P* = 0.005 for the Sarda), this corroborating the hypothesis that the PALMD T allele at c.547 T > A of the accession NM_001035079.1 causes a reduction in the sheep ability to produce milk.

**Table 7:** Average lactation milk yield and days in milk of the analyzed breeds

**Breed**	**Lactation milk yield (l)**	**Days in milk**
Altamurana	41.0 ± 30	133 ± 62
Sarda	152.0 ± 45	239 ± 20

## Discussion

In this study we have performed the genome-wide scan with the Illumina SNP50K Beadchip with the purpose to detect genes which have not yet been annotated in the livestock under investigation. For this reason, the ovine was chosen as target livestock, on one side because of the similarities with cattle in the DNA coding sequences, on the other side because of the abundance of the cattle expressed target sequences in the GenBank data base. Two genes were detected in the genomic regions where highly divergent milk yielders showed different allele frequencies at consecutive markers. These genes may be involved in the variation of milk yield. In fact, PALMD gene codes for a cytosolic protein implicated in membrane dynamics; RFP145 gene codes for an enzyme involved in cellular processes. We also detected novel mutations in these genes: a non-synonymous mutation in exon 5 of the PALMD gene, which was highly correlated with the marker alleles, and could then be assumed as directly responsible of the variation in milk yield. In the RFP gene a non-synonymous mutation was detected in exon 10, as well as two putative binding sites of transcription factors were detected in the 5′UTR and in intron 1. The first is the AT-rich interactive domain factor, member of a protein family provided by helicase and ATPase activities which is thought to regulate transcription of certain genes by altering the chromatin structure around those genes [15]. The second factor, the DM domain-containing transcription factors, is a zinc finger-like DNA binding motif first identified in the sexual regulatory proteins Doublesex (DSX) and MAB-3, and is widely conserved among metazoans [16].

While genomic selection can be still considered the major output of the genome-wide genotyping, we showed in this study a new potential of this strategy: when divergent animals for some traits are genotyped at medium density SNP panel, it is possible to detect novel genes and mutations that might directly affect the trait. This is particularly useful in sheep, where the detection and characterization of genes is a more urgent priority than the genomic selection, because of the lower monetary value of the rams compared to the bulls, and the distribution of the sheep worldwide mainly in lower input production systems.

In this study, the choice of a local sheep breed (the Altamurana) was due to the availability of a good number of milk recorded animals with several consecutive lactations, being this breed maintained for conservation purposes in an experimental farm. Moreover, animals were not submitted to intensive breeding programs and presented a high variability in milk yield within population. The effect of the novel detected mutation on lactation milk yield was later confirmed, independently, in a milk specialized sheep breed.

## Conclusion

In this paper, we present the first genome wide characterization of selective sweeps in sheep breeds with the aim of identifying regions associated with milk production ability. Milk yield is an extremely complex trait, affected by a huge number of genes; in this work, milk yield was chosen as a target trait simply because a population showing an extremely high variability for milk traits was available. Of the nine genomic regions that were made evident in this study as putative candidate regions harboring ovine genes involved in milk performances, only for two of them correspondence was found in the cattle genome with already annotated genes, but this does not imply that the other regions do not encode important genes: it simply means that these genes, to date, have not yet been described. For this reason, the innovation of this study was not the discover of the genes affecting milk production traits, but to show that the DNA genotyping with the Illumina SNP50K Beadchip allowed to detect genes, and mutations in the genes, which have not yet been annotated in the livestock under investigation.

## Methods

### Animal samples

For this study, the Altamurana breed of sheep was selected. This is a local Italian breed of sheep, belonging to the subgroup of south European milk sheep, that lives in a rather harsh environment. The experimental farm on which the animals of the study were raised maintains the local breeds with the aim of conservation and sustainable use of animal genetic resources; sheep were raised using a traditional management system consisting of lambing in November, suckling for 35 d, and then regular machine milking of the ewe twice daily. Animals are not submitted to intensive breeding programs but milk yields and reproductive performances are carefully recorded. Animal management and care followed the recommendations of European Union directive 86/609/EEC.

With the purpose of characterizing selective sweeps for milk yield potential in sheep, this population was chosen for presenting a high variability in milk yield, as well as careful registration of milk recording results for two-three consecutive lactations for each ewe. A group of Sarda sheep, a specialized dairy breed, reared under the same management system and in the same farm as the Altamurana, was included in the analysis in order to validate the obtained results. The average milk yield of the two breeds, considering only the produced milk after the weaning of the lamb, were reported in Table 7, together with the corresponding days in milk.

### Genotyping

A total of 100 sheep of the Altamurana breed were genotyped using the OvineSNP50 BeadChip manufactured by Illumina (San Diego, CA). Genotyping was performed by Porto Conte Ricerche s.r.l. (Alghero - Sassari, Italy). Raw data were analyzed using the GenomeStudio Genotyping Module v. 1.7 (Illumina Inc., San Diego, CA) by applying a no-call threshold of 0.15. File editing was carried out using PLINK [17].

### Statistical analysis of milk data and animal ranking

Milk recording data corresponding to 258 lactations of 100 ewes were used to rank the animals based on the milk potential. The mixed procedure of SAS software [18] was used for this analysis, with the following model:

(1) Yijkl=μ+Bi+Cj+Ak+eijkl

where:

Yijkl = milk yield in the total lactation

B_i_ = fixed effect of the year of lambing

Cj = fixed effect of age of the ewe

A_k_ = random animal effect

eijkl = residual

Similarly to the method of selective genotyping, which consists in genotyping the extreme divergent animals for a trait, the two tails of the distribution of the random animal effect (milk yield residuals) were used to compare the allele frequency at each marker between top and worse milk yielders. The tails were divided in two groups, corresponding to about one sixth (16 sheep) and one twelfth (8 sheep).

The Fisher’s exact test of significance of differences of allele frequency between each pair of the two tails of the distribution was performed for each marker, and statistical significance of the differences between pairs, respectively P_1/6_ and P _1/12_ was considered. It was arbitrarily decided that the interesting markers should satisfy the following condition: P_1/6_ < .05; P _1/12_ <0.001. By requiring the mentioned condition we intended to emphasize that the differences in allele frequencies should become higher as far as the groups became more divergent, as well as that the most divergent group of milk yielders (top and worse twelfth) had highly significant differences in allele frequencies.

Furthermore, it was arbitrarily assumed that when two consecutive markers, where the distance between the two was no more than 100 K bp, satisfied the above mentioned conditions, that region should more likely be harboring a selective sweep.

### Search for the expressed genes in the candidate regions and sequencing of the coding regions

It is useful to compare regions of interest in Ovis aries to the corresponding areas in Bos taurus, as the taurine genome is better annotated [7].

In order to discover if any of the regions implicated in the whole genome analysis contained genes of interest, we used the OAR v3.1 Ovine (Texel) Genome Assembly in http://www.livestockgenomics.csiro.au. After obtaining the ovine sequence of our candidate regions using this browser, the expressed genes were identified by performing a standard nucleotide blast [13] against the NCBI data base of the expressed sequence tags.

Primer pairs to amplify the coding regions of the putative candidate genes were designed on the ovine sequence by using Primer3Plus software (http://www.bioinformatics.nl/cgi-bin/primer3plus/primer3plus.cgi). Direct sequencing of the coding regions of the putative candidate genes was performed on the 3500 Genetic Analyzers (Applied Biosystems, Foster City, CA, USA).

To verify whether any mutations in the 5′ UTR occurred within the putative binding sites for transcription factors, in silico analysis was performed using MatInspector software [14] (http://www.genomatix.de/).

### Assessing the allele substitution effect in independent populations

To verify the hypothesis that novel detected non-synonymous mutations truly affected the target phenotypic trait, average allele substitution effects was estimated in the original Altamurana sample, after direct sequencing of the corresponding amplicon, when DNA of those sheep was still available. Moreover, DNA of 35 sheep of the Sarda breed, raised in the same experimental farm of the Altamurana, with available milk recording results for two lactations, was genotyped, by direct sequencing, at the novel detected non-synonymous mutations. On a data set that contained the lactation yields of the Altamurana and Sarda breeds, the average allele substitution effect of the mutation on milk yield was estimated by a linear regression of the number of alleles (0, 1, or 2), in a linear model that included the breed, the age of the ewe, the year of lambing, and the animal effect [19].

## Competing interests

The authors have no competing interests to declare.

## Authors’ contributions

BM planned the study, extracted the target sequences from the ovine and the cattle assembly, performed the comparisons and drafted the manuscript, MCS performed the direct sequencing, GC provided the milk recording results, FN provided the DNA samples and got them genotyped with the OvineSNP50 BeadChip, RS performed the statistical analysis with GC. All authors have contributed to the editing of the article, and approved the final manuscript.

## Supplementary Material

Additional file 1 Position and potential effects of the detected mutations in the RFP145 gene. Click here for file
