# Supplementary material for: Signatures of selection identify loci associated with milk yield in sheep
Source: BMC Genet. 2013 Sep 3;14:76. doi: 10.1186/1471-2156-14-76 (PMC3844358; doi:10.1186/1471-2156-14-76)
Supplement: Additional file 1 — Position and potential effects of the detected mutations in the RFP145 gene. [file 1471-2156-14-76-S1.doc]

Position and potential effects of the detected mutations in the RFP145 gene

| Location in the gene | Position/accession | Mutation | Putative binding site / amino acid change (1) |  |
| --- | --- | --- | --- | --- |
| 5’ UTR | 108 bp upstream transcription initiation | C/T | ggagcttttTACAttataaca | DM domain-containing transcription factors |
| Intron 1 | 37 bp downstream exon 1 | C/T | tcagaATATtcactaaaagct | AT rich interactive domain factor |
| Exon 2 | NM_001102168 | c.551C>T | Synonymous (Leu/Leu) |  |
| Exon 4 | NM_001102168 | c.823C>/T | Synonymous (Leu/Leu) |  |
| Exon 10 | NM_001102168 | c.2414G>A | Lys/Glu |  |

1. The mutation position is underlined
